# Microbial analysis of *Zetaproteobacteria* and co-colonizers of iron mats in the Troll Wall Vent Field, Arctic Mid-Ocean Ridge

**DOI:** 10.1371/journal.pone.0185008

**Published:** 2017-09-20

**Authors:** Jan Vander Roost, Ingunn Hindenes Thorseth, Håkon Dahle

**Affiliations:** 1 Centre for Geobiology, University of Bergen, Bergen, Norway; 2 Department of Biology, University of Bergen, Bergen, Norway; 3 Department of Earth Science, University of Bergen, Bergen, Norway; National Cheng Kung University, TAIWAN

## Abstract

Over the last decade it has become increasingly clear that *Zetaproteobacteria* are widespread in hydrothermal systems and that they contribute to the biogeochemical cycling of iron in these environments. However, how chemical factors control the distribution of *Zetaproteobacteria* and their co-occurring taxa remains elusive. Here we analysed iron mats from the Troll Wall Vent Field (TWVF) located at the Arctic Mid-Ocean Ridge (AMOR) in the Norwegian-Greenland Sea. The samples were taken at increasing distances from high-temperature venting chimneys towards areas with ultraslow low-temperature venting, encompassing a large variety in geochemical settings. Electron microscopy revealed the presence of biogenic iron stalks in all samples. Using 16S rRNA gene sequence profiling we found that relative abundances of *Zetaproteobacteria* in the iron mats varied from 0.2 to 37.9%. Biogeographic analyses of *Zetaproteobacteria*, using the ZetaHunter software, revealed the presence of ZetaOtus 1, 2 and 9, supporting the view that they are cosmopolitan. Relative abundances of co-occurring taxa, including *Thaumarchaeota*, *Euryarchaeota* and *Proteobacteria*, also varied substantially. From our results, combined with results from previous microbiological and geochemical analyses of the TWVF, we infer that the distribution of *Zetaproteobacteria* is connected to fluid-flow patterns and, ultimately, variations in chemical energy landscapes. Moreover, we provide evidence for iron-oxidizing members of *Gallionellaceae* being widespread in TWVF iron mats, albeit at low relative abundances.

## Introduction

Biological communities in hydrothermal systems are driven by chemotrophic primary producers, utilizing the energy available in chemical disequilibria, forming when reduced hydrothermal fluids mix with oxic seawater. The geological and geochemical setting varies between and within hydrothermal systems, giving rise to shifting chemical energy landscapes [1, 2]. Revealing how this variation shapes the distribution of functional groups of microorganisms is not only important for understanding the microbial ecology of hydrothermal systems, but also for our general understanding of how geochemistry is linked to microbiology. Hydrothermal systems are considered to play a significant role in the marine iron cycle, with estimated releases of iron in the order of 50 Gg year^-1^ [3]. Recent analyses indicate that a substantial part, if not most, of this iron is released by slow, diffuse, low-temperature venting as opposed to focused, high-temperature venting through chimneys [4–7]. Iron cycling involves iron-oxidizing bacteria (FeOB), which obtain energy from the oxidation of ferrous iron (Fe(II)) to ferric iron (Fe(III)) [8]. Under anoxic conditions in the subseafloor, iron is stable in its reduced form. However, when Fe(II) mixes with seawater, it oxidizes abiotically and precipitates as iron oxyhydroxides. FeOB are therefore typically found in habitats with low oxygen concentrations, where there is less competition from abiotic iron oxidation [8]. The first representatives of *Zetaproteobacteria* were discovered by molecular methods from studies of the Loihi Seamount [8, 9]. Since then, members of this group have been detected in various marine environments around the world, such as iron mats in hydrothermal fields, basalt, steel corrosion enrichment experiments and the brine-seawater interface [10–13]. Yet, *Zetaproteobacteria* seem to be restricted to iron-rich habitats and cultured representatives within this class are all lithotrophic iron oxidizers that produce micrometre-scale iron oxyhydroxide particles with characteristic morphologies that can be recognized by light microscopy [5, 14, 15]. Molecular studies indicate that *Zetaproteobacteria* show a strong biogeographic signal with endemic, phylogenetic clades, at least within different regions of the Pacific Ocean [11]. However, little is known about intra-field variations regarding relative abundances of *Zetaproteobacteria* and their co-occurring taxa, and the factors that control this variability. This is particularly true for crustal spreading zones, where molecular evidence for the presence of *Zetaproteobacteria* has been scarce [14, 16].

The Arctic Mid-Ocean Ridge (AMOR), located at the northern part of the Mid-Atlantic Ridge (MAR) hosts the Jan Mayen Vent Fields (JMVFs), which consist of two neighbouring hydrothermal systems: the Troll Wall Vent Field (TWVF) and the Soria Moria Vent Field [16]. At the TWVF, numerous iron deposits at areas of diffuse low-temperature (2–7°C) venting, cover an area of at least 104 m^2^ [17]. These deposits, which are found some hundreds of metres away from high-temperature (≤270°C) venting chimneys, vary in size and shape from cm-thick mats to several metre high mounds. So far, the microbial community structure has been analysed in the surface layer of one iron mound, demonstrating a dominance of *Zetaproteobacteria* [17]. This stands in contrast to the white bacterial mats dominated by sulphur-oxidizing bacteria (SOB), typically found near the base of the high-temperature smokers [18]. The aim of the present study was to reveal the underlying reason for this shift in community composition and to infer what effect variations in geochemical setting, and hence energy landscapes, has on relative abundances of *Zetaproteobacteria* in TWVF. Moreover we wanted to assess the uniqueness of phylogenetic clades of *Zetaproteobacteria* from this area. To this end we analysed five iron mats from variable geochemical settings at variable distance from the high-temperature venting chimneys. From our results we infer that variations in fluid flow patterns give rise to energy landscapes with shifting densities of iron oxidation energy, which in turn, largely shapes the distribution of FeOB.

## Materials and methods

### Site description

**Fig 1:**
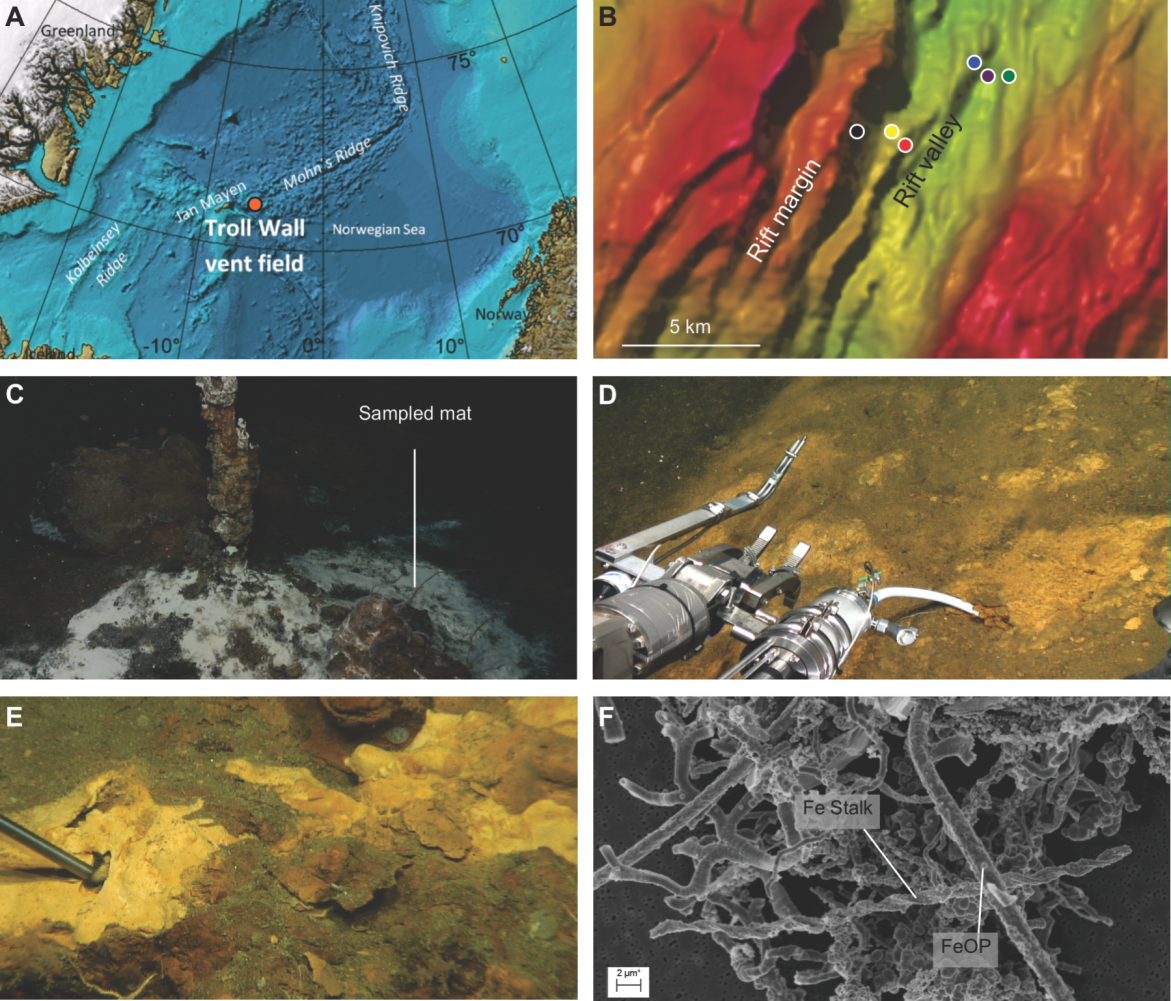
Location of the TWVF and images of sampling sites and samples. (A) Location of the Troll Wall vent field at MAR. The figure was adapted from the GEBCO Digital Atlas, published by the British Oceanographic Data Centre on behalf of IOC and IHO, 2003. (B) Bathymetric map indicating sampling locations at the rift margin (yellow = 11ROV3, red = 12ROV5, black = previously published TWVF white mat [18]) and rift valley (purple = 11ROV6, blue = 12ROV9, green = 14ROV13). (C) Sampling location of the white microbial mat studied in [18]. (D) Iron mat sampling at the rift margin (11ROV3). (E) Iron mat sampling at the rift valley (12ROV9). (F) SEM image of TWVF iron mats showing dense networks of iron stalks intertwined with numerous elongated deposition structures; i.e. non-branching vs. branching, hollow vs. solid and curled vs. straight filaments. FeOP, Fe-oxyhydroxide particles.

The TWVF, part of JMVFs (Fig 1A), harbours a large volcano, transected by a 150 m deep rift valley (71°N, 6°W) (Fig 1B). The rift valley (700 mbsl) is covered by numerous rust-colored microbial mats at sites of ultra-diffuse venting, as indicated by moderate temperature gradients with temperatures of 0–5°C at 20 cm below seafloor. Local microbial communities reside on a substrate primarily composed of silicified hyaloclasite and basaltic debris. More active venting takes place at the eastern rift margin (550 mbsl). Here, focused fluid flow occurs through white smoker hydrothermal chimneys, composed of anhydrite, barite and pyrite. The chimneys are surrounded by patches of white microbial mats [17, 18]. The macrofauna present at TWVF consists mostly of calcareous sponges, sea anemones and sea stars [19].

### Sampling

Five iron mats (Fig 1), comprising 13 discrete samples in total (S1 Table), were collected from the TWVF during cruises with R/V G.O. SARS between 2011 and 2014. Samples were taken with a remotely operated vehicle [20] equipped with a 1L hydraulic suction pump (‘biosyringe’). Parallel samples from each iron mat were subjected to independent DNA extractions and generation of 16S rRNA gene amplicons. As a reference, a background seawater sample (11ROV11) was collected 50 m above the seafloor (approximately 500 mbsl) and approximately 100 m away from active, high-temperature venting.

### Chemical analysis

The pH and alkalinity of the fluids in the biosyringes were measured shipboard immediately after sampling by a portable pH-meter and an autotitrator (Titrando 888), respectively. Ammonium, nitrate+nitrite, and phosphate were measured photospectrometrically by a Quaatro continuous flow analyser (Seal), directly after sampling. Aliquots for later analyses of major and minor elements by inductively coupled plasma optical emission spectrometry (ICP-OES) (Thermo elemental IRIS) were acidified to 3 vol% of ultrapure HNO_3_ and stored in acid-cleaned HDPE bottles. Both aliquots for ICP-OES and aliquots for anion analysis by Ion Chromatography (Metrohm) were stored at 4°C until onshore analyses.

### Scanning electron microscopy

Aliquots of iron mat samples for scanning electron microscopy were fixed in 2% glutaraldehyde. Dehydration steps were performed on a 0.2 μM pore filter and exposed to, sequentially, 50%, 75% and three times 100% ethanol solutions, with a settling time of 15 minutes between each step. Samples were glued to Al-stubs and coated with Ir using a Gatan 682 Precision etching coating system. C-coating was done with an Agar Turbo carbon coater. Images were taken with a Zeiss Supra 55VP Field Emission Scanning Electron Microscope, equipped with a Thermo Noran Six Energy Dispersive Spectrometer system.

### DNA extraction, 16S rRNA amplification and 454 sequencing

DNA was extracted from 600 μL sample material with the FastDNA^®^ Spin Kit for soil (MP Biomedicals, Solon, OH) using the manufacturer´s instructions. Cells were lysed in a FastPrep instrument (MP Biomedicals, Santa Ana, CA) at a speed setting of 6.0 for 40 seconds. The 16S rRNA gene amplifications were generated with primers universal for *Bacteria* and *Archaea*: Uni787F (5’-ATTAGATACCCNGGTAG-3’) [21] and Uni1391R (5´-ACGGGCGGTGWGTRC-3’) [22]. PCR was performed with 12.5 μL 2x HotStar Taq master mix (Qiagen), 1 μM of each primer, 1 μL DNA template and ddH_2_O to a total volume of 25 μL. Reactions were run with the following program: (5’ 95°C), 30 X [(45” 95°C) (45” 53°C) (1’ 72°C)], (7’ 72°C). 16S rRNA gene fragments were evaluated on size on a 1.5% agarose gel. DNA samples were purified from PCR reagents with the MinElute PCR purification kit (Qiagen) following the manufacturer´s protocol and eluted in 15 μL ddH_2_O. A second PCR of 7 cycles was carried out to tag amplicons with barcodes, using 20 μL reactions with 1 μM of each primer, 10 μL 2x HotStar Taq master mix (Qiagen), and 0.8 ng of template. Finally 16S rRNA tagged fragments were purified by AMPure XP Bead Purification (Agencourt) following manufacturer´s instructions. DNA concentrations were quantified using a Bioanalyzer (Agilent Biosystems). Equivalent amounts of DNA per sample were pooled prior to sequencing with Multiplex GS FLX+/Titanium 454 pyrosequencing technology (Roche) at the Norwegian High-Throughput Sequencing Centre in Oslo (NHS), Norway and at Microsynth in Balgach, Switzerland.

### Sequence data processing and OTU analysis

Raw sequence data was processed with MOTHUR (version1.33.2) using a pipeline based on the publicly available 454 SOP as accessed on 11/11/2015 (http://www.mothur.org/wiki/454_SOP) [23,24]. Filtering of sequences was performed with Ampliconnoise as implemented by MOTHUR (‘shhh-flows’ command). After removal of barcodes and primer sequences, all reads were merged and aligned to a SILVA reference alignment database (silva.nr_v119.align). The default Needleman-Wunsch aligning algorithm was applied, with k-mer template searching and using +1 per match and mismatch penalties -1, -2 and -1 for each mismatch, opening and extension of a gap, respectively. The alignment was cropped to a minimum length of 210bp and further optimized by selecting both start and end positions by which 90% of the sequences started or ended. Sequences with homopolymers longer than 6 bp were removed. Further filtering and preclustering of sequencing data was carried out as described in [24]. Chimeras were removed from the dataset using default settings of the UCHIME program [25] as implemented in MOTHUR. More detailed information on the filtering of reads is given in the S2 Table.

Classification of reads and clustering into operational taxonomic units (OTUs) at 97% sequence similarity was carried out as described in [24]. A total of 5573 OTUs were identified of which 1877 remained after removal of single- and doubletons. Rarefaction plots were generated using the MOTHUR command ‘rarefaction.single’. In addition, richness [26] and diversity (inverse Simpson) indices were generated, with the ‘summary.single’ command.

Non-metric multi-dimensional scaling (NMDS) plots were generated using the Vegan package in R [24] and were based on Bray-Curtis distances, obtained using the average neighbour clustering algorithm. Analysis of similarities (ANOSIM) [27] was used to assess the significance of differences between microbial mats from the rift valley and the rift margin, in which the R-test statistic provides a measure for the separation of community structures; with R = 0 designating no separation, R < 0.25 as barely separable, R > 0.5 as separated but overlapping and R > 0.75 indicating well-separated community structures [28]. Heatmaps showing the compositional profile of the different iron mat communities were generated in R with the ‘heatmap.2’ function within the gplots package version 2.11.0.1 [29] where samples (columns) were clustered hierarchically, by complete linkage with Euclidean distance measure, and OTUs (rows) clustered phylogenetically, by incorporating a phylogenetic tree from MEGA version 5.2.2. This neighbour-joining tree, comprising the 50 main OTUs, was built with default settings of the incorporated maximum composite likelihood algorithm; using default ClustalW parameters (gap opening penalty = 15 and gap extension penalty = 6.66).

To contribute to the effort of mapping the global distribution of *Zetaproteobacteria*, all *Zetaproteobacteria* reads from TWVF were assigned to predefined OTUs (ZetaOtus), using the curated ZetaHunter application (https://github.com/mooreryan/ZetaHunter, last accessed 20/06/2017) [11]. ZetaHunter uses the SILVA v123 phylogenetic reference database in order to assign query sequences to ZetaOtus [23, 25, 30–32]. For comparison, all MAR sff-files provided by [14] were reanalysed using MOTHUR and ZetaHunter as described above. Due to differences in the targeted 16S rRNA gene region, we were not able to directly compare *de novo* ZetaOtus constructed for TWVF with those constructed from the other MAR samples.

### Data submission

Original sff-files were submitted to the European Nucleotide Archive with study accession number PRJEB11309, sample accession numbers ERS903619-39, experiment accession numbers ERX1135963-83, and run accession numbers ERR1055648-68.

## Results

### Environmental settings, estimated cell numbers, and electron microscopy

Sampling sites close to the TWVF white smokers at the eastern rift margin (11ROV3 and 12ROV5), were characterized by higher temperatures, lower pH, higher alkalinity and lower concentrations of Fe^2+^, SO_4_^2-^, NO_3_^-^ and PO_4_^3-^, compared to sites in the ultra-slow, diffuse-flow systems in the central rift valley (11ROV6, 12ROV9 and 14ROV13) (S1 Table) (Fig 1C and 1D). Estimated cell numbers from DNA extraction yields are similar to cell numbers reported for iron mats at MAR [14], and were 1–2 orders of magnitude higher in the iron mats than in background seawater (S1 Table). Using electron microscopy, we identified twisted stalks as well as Fe-rich particles with uncharacteristic biological morphologies in all mats (Fig 1E and 1F).

### Community structures and diversity

**Fig 2:**
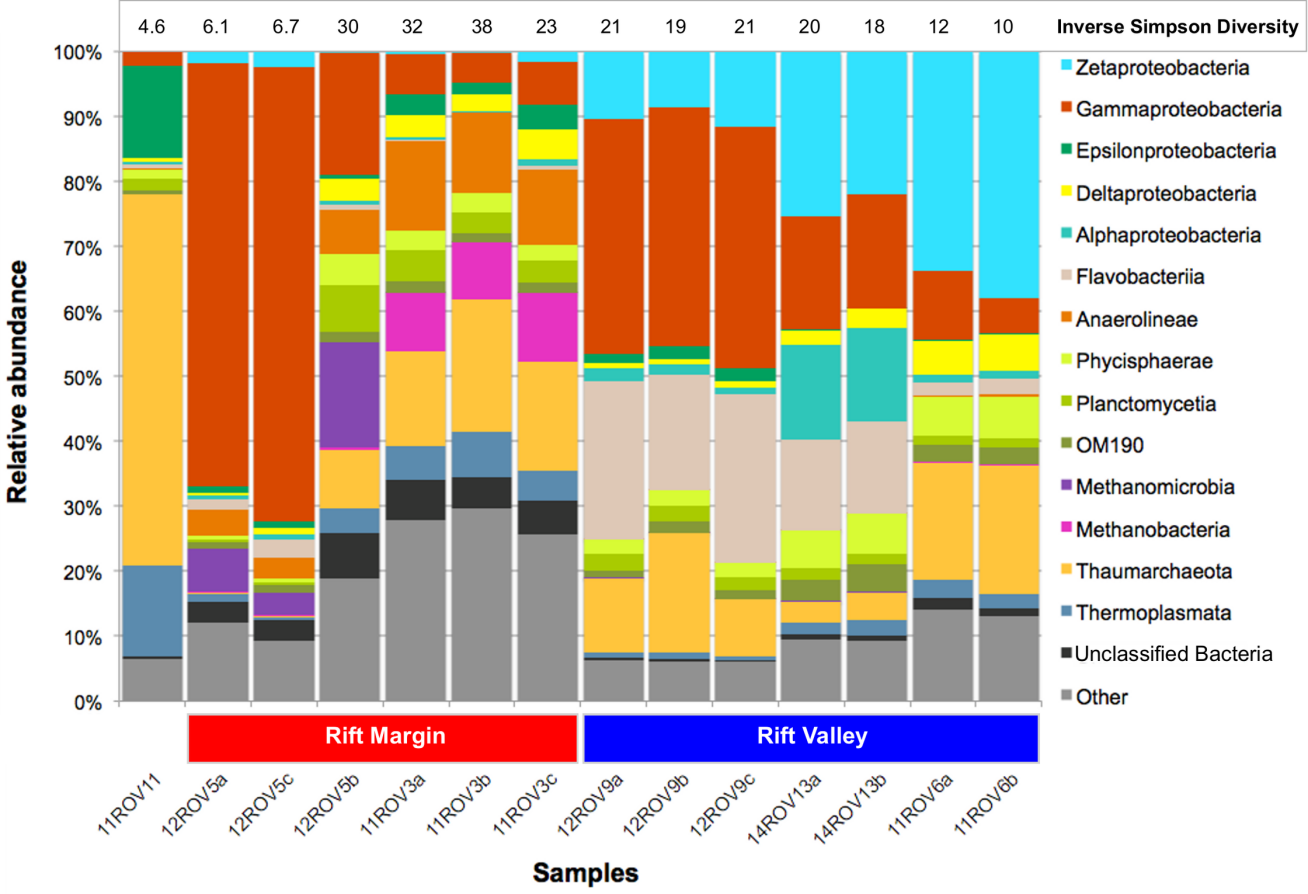
Class-level taxonomic composition of iron mat communities. Numbers above each bar represent inverse Simpson diversity indices for each sample. ‘Unclassified Bacteria’–Bacteria not classified on class-level. ‘Other’–All sequences not falling into any of the listed taxonomic groups.

**Fig 3:**
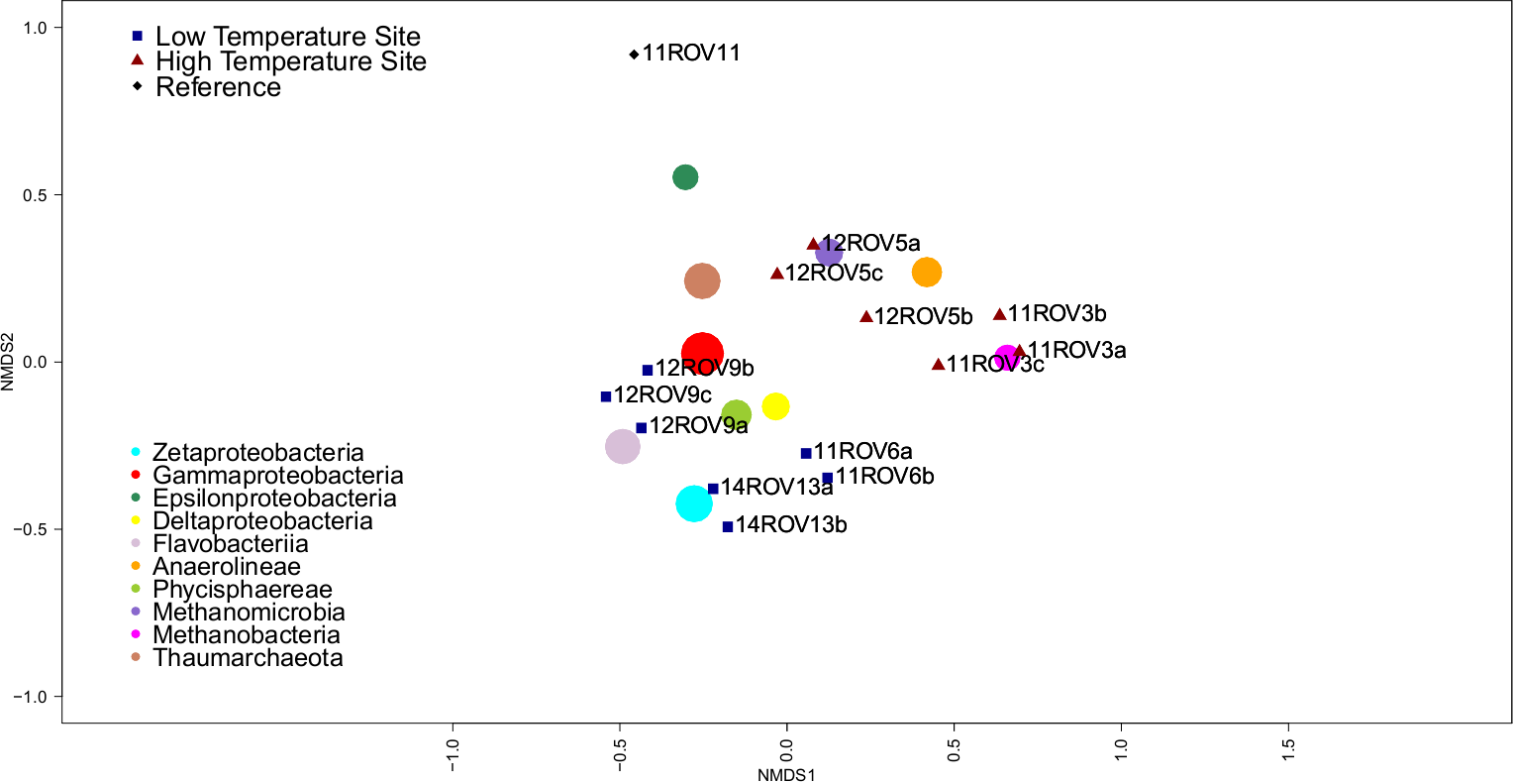
NMDS plot of the iron mat communities on class level. Blue squares indicate samples from the rift valley and red triangles indicate samples from the rift margin. Dots indicate major microbial classes and have a radius proportional to overall relative abundance. See S3 Fig for a plot based on OTU-level comparisons.

**Fig 4:**
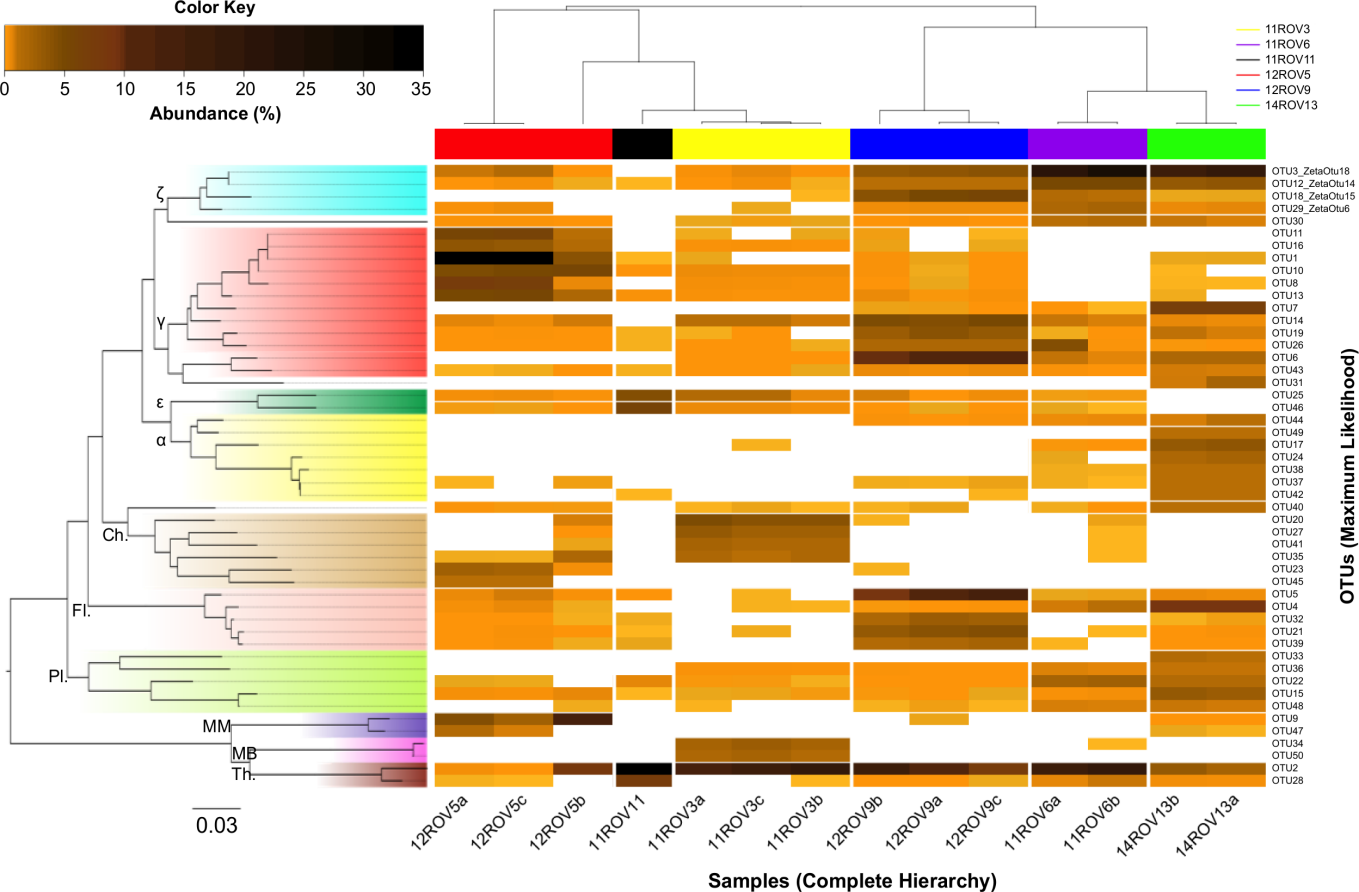
Heatmap showing the distribution of the 50 most abundant OTUs (>0.05% total relative abundance) within the sampled iron mats. Abundances range from low (yellow) to high (dark brown), and absent OTUs are shown as white. Samples are clustered by complete hierarchical clustering (x-axis) and OTUs are grouped phylogenetically (y-axis) by the neighbour-joining algorithm. ζ = *Zetaproteobacteria*, γ = *Gammaproteobacteria*, ε = *Epsilonproteobacteria*, α = *Alphaproteobacteria*, Ch. = *Chloroflexi*, Fl. = *Flavobacteria*, Pl. = *Planctomycetes*, MM = *Methanomicrobia*, MB = *Methanobacteria*, Th. = *Thaumarchaeota*.

Considering all iron mats, six phyla dominated (>1% total relative abundance); *Proteobacteria* (43.1%), *Thaumarchaeota* (16.7%), *Bacteriodetes* (9.6%), *Planctomycetes* (8.5%), *Euryarchaeota* (7.4%) and *Chloroflexi* (6.7%). However, we observed a large variability in class-level community structure between the different iron mats (Fig 2). In an NMDS ordination based on Bray-Curtis distances, we found that parallel samples from the same iron mat formed distinct clusters (Fig 3). Furthermore, Analysis of Similarities (ANOSIM), revealed a significant separation between rift valley samples and rift margin samples (R-value = 0.3201, p-value = 0.006). *Zetaproteobacteria* were observed in all samples, but reached the highest abundances (37.9%) in the rift valley (Figs 2 and 3). Rift margin samples were characterized by high abundances of *Anaerolinae* as well as *Methanomicrobia* (11ROV3) and *Gammaproteobacteria* (12ROV5). Most of the *Gammaproteobacteria* detected in 12ROV5 were further assigned to *Methylococcales*, whereas most of the *Gammaproteobacteria* in other mats were *Alteromonadales*, *Oceanospirillales*, *Marinicellales*, or not assigned on the order level. The most abundant *Epsilonproteobacteria* OTUs (OTUs 25 and 46), were relatives of sulphur-oxidizing genera, and closely related to *Sulfurimonas autotrophica* (97%) and *Sulfurovum lithotrophicum* (94%), respectively. They were found in all iron mats except for the 14ROV13 samples. *Deltaproteobacteria* were present with similar relative abundances across the different mat samples, and were mostly assigned to genera comprising sulphate reducers—i.e. *Desulfuromonadales* and *Desulfarculales* (Fig 2, S1 Fig). Most *Thaumarchaeota* (90%) were assigned to the genus *Nitrosopumilus*, which comprises ammonium oxidizers [33]. In order to identify patterns on a OTU-level resolution, we looked at variations in relative abundances of individual OTUs (Fig 4). This revealed some variation in the presence and abundance among OTUs assigned to the same class. For example, among *Zetaproteobacteria*, 12ROV9 was dominated by OTU18, as opposed to all other mat samples, which were dominated by OTU3 or OTU12. These latter two OTUs were both 98% similar to the 16S rRNA gene of the iron-oxidizing *Mariprofundus ferrooxydans* PV-1. OTU18 was only 94% similar to OTU3 and 12, but showed 100% sequence identity with a sequence obtained from the Levantine continental margin [KF199324], a site without hydrothermal activity, where ferrous iron originates from turbation of soft sediments at a depth of 800 mbsl [34]. Notably, the most dominating OTU in the background seawater, assigned to the *Nitrosopumilus* genus, was also dominating among *Thaumarchaeota* in the iron mat samples. From rarefaction analyses (S2 Fig) and Shannon-index values (Fig 2), we found that the 11ROV3 samples had the highest diversity and that the diversity in all mat samples was higher than in background seawater.

### Mapping of TWVF *Zetaproteobacteria* to ZetaOtus

**Fig 5:**
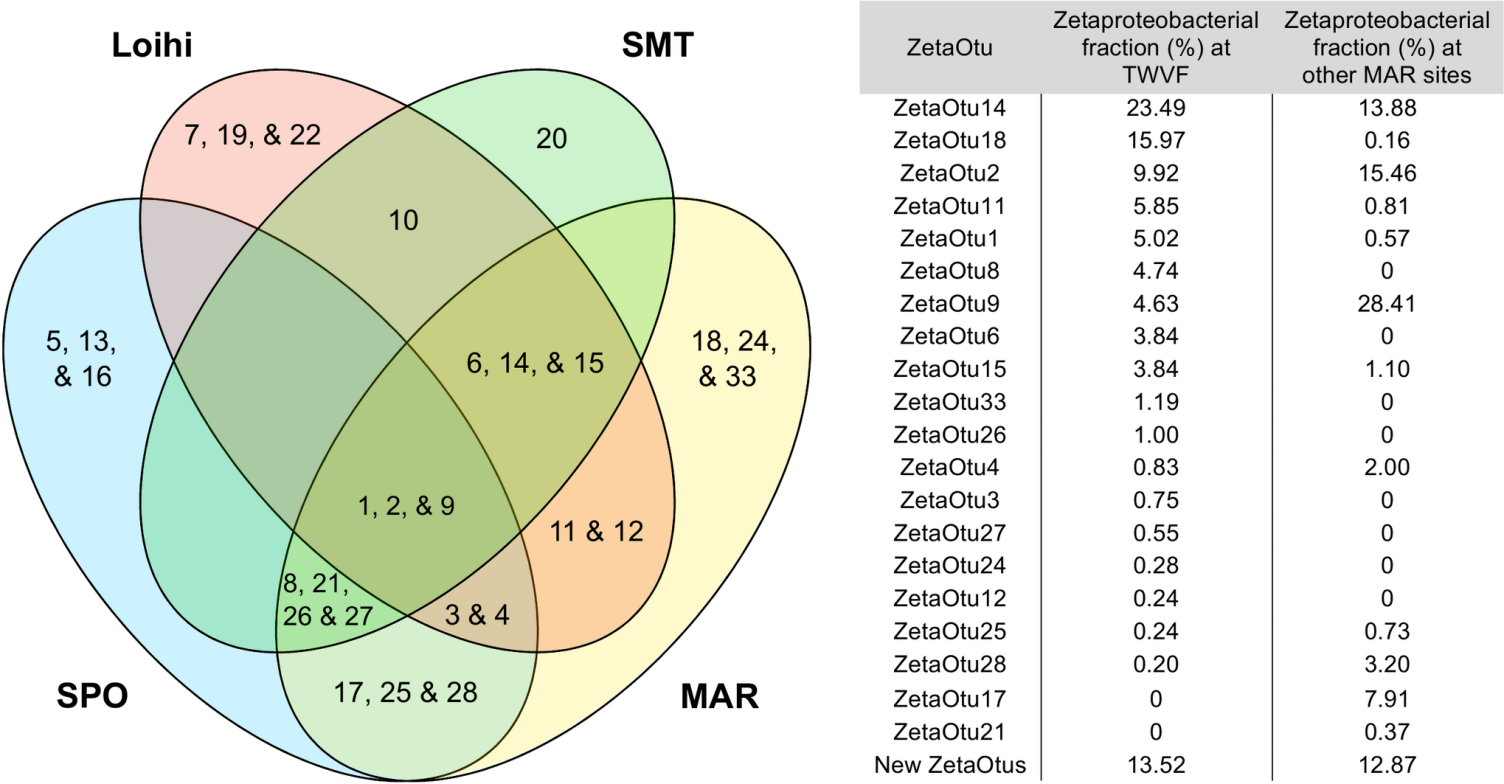
The distribution of Zetaproteobacterial OTUs (ZetaOtus) at Loihi Seamount, Southern Mariana Trough (SMT), South Pacific Ocean (SPO) (Vailulu’u Seamount, Tonga Arc, East Lau Spreading Center, Kermadec Arc) and Mid-Atlantic Ridge (MAR) (TWVF, Snake pit, Rainbow, TAG). The figure is based on ZetaHunter analysis of MAR sequences (This study, [14]), as well as information from [11, 35].

Using ZetaHunter, we identified 18 different ZetaOtus in the TWVF samples. This includes ZetaOtus found in geographically separated environments, such as the Loihi Seamount, the South Pacific Ocean (Vailulu’u Seamount, Tonga Arc, East Lau Spreading Center, Kermadec Arc), the Mariana Trough, and the Mid-Atlantic Ridge (MAR) (Fig 5). Among these, ZetaOtus 1, 2, and 9 are considered as cosmopolitan [11, 35]. It should also be noted that ZetaOtu18, the most dominant ZetaOtu specific to MAR/TWVF in Fig 5, has previously been detected at the Juan de Fuca ridge in the Pacific and in the Gulf of Maine [11, 12]. ZetaOtu24, detected at TWVF but so far undetected at other MAR sites, also includes Guaymas Core B clone B03R022 [AY197408] from the Gulf of California [36]. Finally, ZetaOtu33 includes clone T13J-B63 [JNH860378] obtained from the Southwest Indian Ridge [11, 37]. Using ZetaHunter, 22 *de novo* OTUs were constructed from all TWVF samples. However, individual relative abundances of these Zetaproteobacterial OTUs were low (<1.7%). Hence, none of the most dominating ZetaOtus were found to be unique for TWVF. The four most abundant OTUs assigned to *Zetaproteobacteria* in the ZetaHunter-independent OTU clustering, corresponded to ZetaOtus 18, 14, 15 and 6 (Fig 4).

### Functional assignments

Based on taxonomic information and extensive BLAST searches, we inferred functions of OTUs closely related to cultured organisms (S3 Table). From this, we constructed functional-level community structures, indicating how the distribution of functional groups varied between different mat samples (S1 Fig). In these analyses we also included a previously published dataset from a microbial mat located at the base of one of the white smokers in TWVF [18], and which is a typical representative of the numerous white mats observed by ROV video-imaging in this region (not shown). This analysis indicated a functional zonation in samples taken at increasing distances from the white smokers towards the central rift valley. Whereas white mats at the base of the high-temperature venting chimneys are dominated by sulphide oxidizers and to some extent methane oxidizers, iron mats in the rift margin harbour high abundances of methanogens and (for 12ROV5) methanotrophs. Finally, the rift valley iron mats are characterized by high abundances of putative iron oxidizers.

### Distribution of *Gallionellaceae*

Relative abundances of *Gallionellaceae* varied between 0–0.5% in the iron mats. Within *Gallionellaceae*, the same three OTUs dominated in all samples (OTUs 75, 344 and 2013), except for 1 subsample of the 12ROV5 dive from the rift valley where *Gallionellaceae* were not detected. These three OTUs were all 97–98% similar to *Gallionella* sp. JA52 [KC677661.1] [38], derived from a mine water treatment plant (Freiberg, Germany). In order to evaluate whether the detected Gallionellacea*e* represented contaminants introduced during DNA extraction or PCR, we compared relative abundances of *Gallionellaceae* with estimated cell numbers. We reasoned that if the *Gallionellaceae* represented contaminants, they would have a tendency to occur with higher abundances in samples with low DNA yields (i.e. samples with low estimated cell numbers). However, no such correlation was observed. Instead, we observed a correlation between relative abundances of *Gallionellaceae* and *Zetaproteobacteria* (R^2^ = 0.7845, p-value = 8.917E-4) (S4A Fig), which provides evidence that *Gallionellaceae* are indeed indigenous to the mats, with relative abundances controlled by the same environmental factors as for *Zetaproteobacteria*.

## Discussion

**Fig 6:**
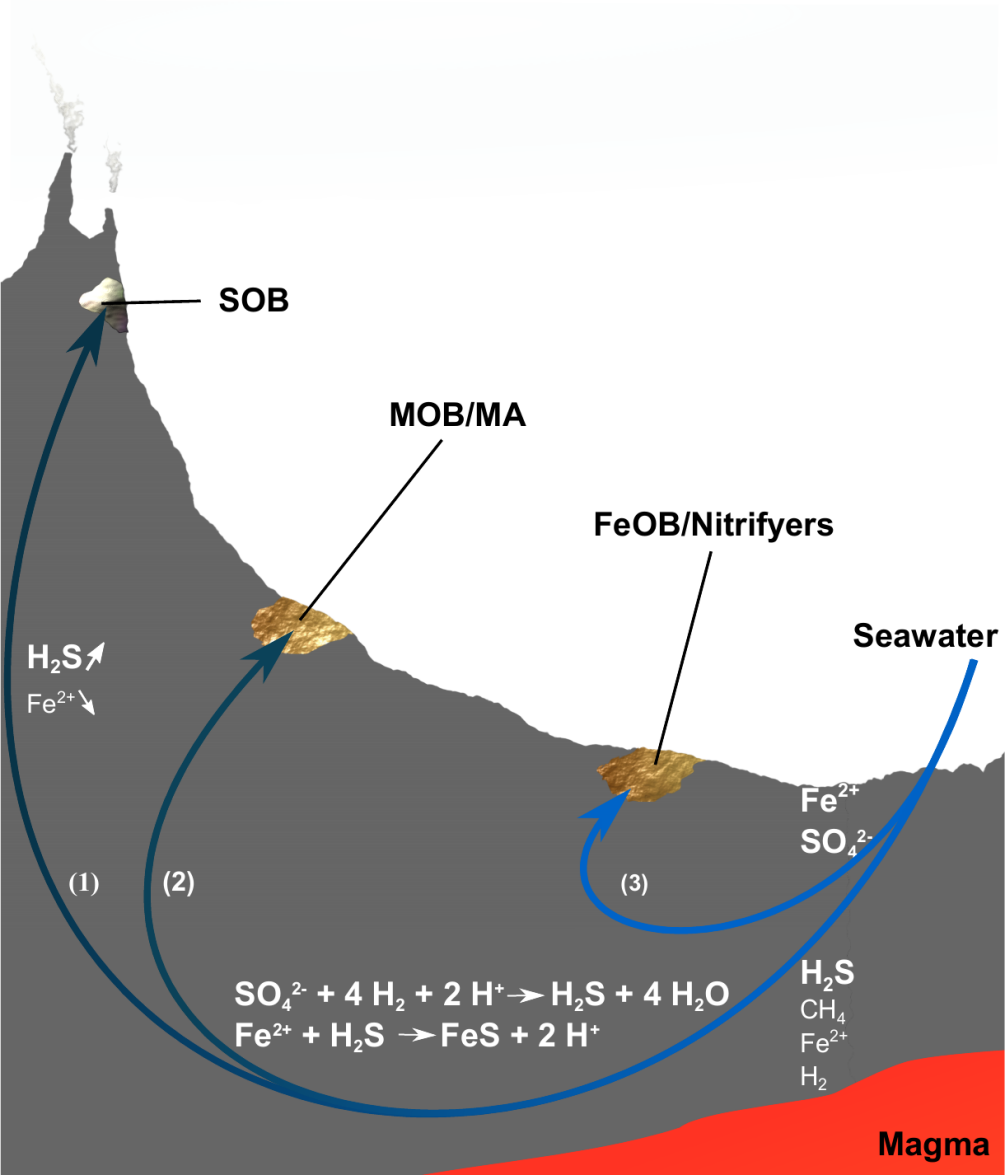
Proposed model on how fluid flow patterns and geobiological factors shape energy landscapes and microbial community composition in different regions within the TWVF. (1) High-temperature fluids venting through the chimneys and flowing through sediments in the vicinity of the chimneys are formed deep inside the crust and have high concentrations of sulphide as well as moderate concentrations of methane and hydrogen. Iron concentrations are low due to precipitation of pyrite inside the crust. White microbial mats are abundant on and around the active chimneys and have relative abundances of sulphide oxidizers and methane oxidizers that are consistent with communities predicted from energy models considering mixing of high-temperature fluids with seawater [1]. (2) Rare-earth element analyses suggest that hydrothermal fluids flowing through iron mats in the rift margin, have the same source as the high-temperature fluids flowing through the chimneys (not published). However, subseafloor precipitation of sulphides lead to conditions where Fe(II), leaching out from minerals or produced by iron reducers, is more stable and forms the basis for the development of iron deposits inhabited by low abundances of FeOB on the seafloor. Yet, high abundances of methanotrophs and methanogens are indicative of a microbial degradation of organic matter with organics as the main source of energy under this setting. Accumulation of organic carbon in the rift margin may partly be a result of transport of biomass from the most active hydrothermal areas down towards the rift valley. (3) As suggested by rare-earth element analysis, fluids circulating through the rift valley belong to a shallow circulation system that is separated from the high-temperature fluids [17]. Sulphate is therefore not reduced to sulphide abiotically, and lower amounts of Fe(II) precipitate in the crust. This forms the basis for conditions on the seafloor with relatively high densities of potential energy from iron oxidation, as reflected by communities with high relative abundances of FeOB. SOB = sulphur-oxidizing bacteria, MA = methanogenic archaea, MOB = methanotrophic bacteria, FeOB = iron-oxidizing bacteria.

Using 16S rRNA gene amplicon sequencing data we have investigated shifts in seafloor microbial mats from the Troll Wall Vent Field in environments variable in terms of hydrothermal activity and distance from high-temperature venting smokers. With cell numbers 1–2 orders of magnitude lower in the background seawater than in the iron mats, the influence of iron mats by background seawater can be expected to be minor. Moreover, the high similarity between replicates indicates that the observed differences between iron mats are not only an effect of biases in DNA extraction and PCR amplification, but reflect real variation. *Zetaproteobacteria* were detected in all iron mat samples, indicating that this class is widespread at TWVF. However, relative abundances of iron oxidizers and their co-occurring taxa seem to vary largely among sites. Below we compare these findings with other iron mats and discuss a potential metabolic interaction between members of *Zetaproteobacteria* and members of *Nitrosopumilus*. Based on geochemical data from TWVF fluids, we discuss how the observed transition from microbial mats dominated by sulphur oxidizers near hydrothermal chimneys, to microbial mats dominated by iron oxidizers in the rift valley, might be linked to shifting fluid-flow patterns and, ultimately, shifting energy landscapes. This is summarized in a conceptual model of the geobiological interactions taking place at the TWVF (Fig 6).

### Comparison with iron mats from other locations

On the taxonomic level, the microbial iron mats analysed in this study share some of the characteristics of other iron mats around the world–i.e. the presence of close relatives of sulphate reducers within *Deltaproteobacteria* [39–41], the presence of *Epsilonproteobacteria* [42, 43], a high abundance of close relatives of ammonium-oxidizing *Nitrosopumilus* among *Archaea* [40, 41, 43], and the presence of *Chloroflexi* [39, 41]. Yet, to our knowledge, this is the first report indicating that methanogens may make out a large fraction of microorganisms in iron mats. Members of methanogens and sulphate reducers are strict anaerobes, hence their presence is likely to reflect steep oxygen gradients at the sampling sites. Presumably, obligate anaerobes grow in the lower parts of the iron mat or in the transition from iron mat to the underlying sediments, and arguably contribute to the production of methane and hydrogen sulphide, consumed by aerobic methanotrophs and sulphide oxidizers in upper layers.

With respect to marine iron oxidizers, insights into the biogeographical patterns of *Zetaproteobacteria* have been documented in several recent papers [11, 44–47]. On a global level, the distribution of *Zetaproteobacteria* OTUs has been found to be more strongly correlated with geographic occurrence than with environmental parameters such as temperature, pH, or total Fe concentration [11, 48]. Our results are in agreement with this in the sense that many of the same OTUs of *Zetaproteobacteria* seem to dominate in both rift margin as well as rift valley samples. Hence, shifting geological settings between the iron mats (see below) seem to have a large effect on relative abundances of *Zetaproteobacteria* and what taxa they co-occur with, but without causing large shifts in what OTUs of *Zetaproteobacteria* the different mats host. Moreover, our ZetaHunter analyses (Fig 5) did not reveal any new dominating ZetaOtus specific to TWVF. Similar observations were made from the Snake Pit, Rainbow and TAG site of the Mid-Atlantic Ridge [14], suggesting that *Zetaproteobacteria* from the Atlantic region are highly similar to their relatives from around the world and probably do not constitute any distinct phylogenetic clades. The detection of ZetaOtus 1, 2 and 9 supports earlier findings that these ZetaOtus might be cosmopolitan [11, 35]. Recently, it was suggested that ZetaOtu1 may not be truly cosmopolitan, due to its low abundance at MAR sites [35]. However, ZetaOtu1 was identified as one of the dominating ZetaOtus at TWVF (Fig 5) demonstrating that this ZetaOtu may also be found in high abundances at MAR. High abundances of ZetaOtus 6 and 14 at TWVF are also inconsistent with the recent suggestion that these ZetaOtus could be considered as geographic biomarkers for the Loihi Seamount. The comparison between TWVF and other MAR sites demonstrates that the distribution of ZetaOtus can differ significantly between geographically distant MAR sites (Fig 5). For example, ZetaOtu18 is the second most abundant ZetaOTU at TWVF, but barely present elsewhere on MAR. Taken together, these findings illustrate that our knowledge about the global distribution of ZetaOtus, including those from MAR sites, is still too limited to draw strong conclusions on biogeographical patterns. It is also too early to exclude the possibility of geochemical setting being the main driver for shaping communities of *Zetaproteobacteria* worldwide.

The presence of *Gallionellaceae* in all iron mat samples is noteworthy. This group is traditionally considered to consist exclusively of freshwater iron oxidizers. Although some recent studies report on the presence of this group in marine environments [42, 49–53], their relative abundance has been so low (<0.1%) that it has remained unclear if they represent contaminants or not. Low abundances of *Gallionellaceae* were also observed in the present study. However, if they represented contaminants of some sort, one would expect a negative correlation between DNA content in the samples and relative abundances of *Gallionellaceae* and no relationship between relative abundances of this family and other taxa. Our results indicate that the opposite is true: we found no correlation between estimated cell numbers and relative abundances of *Gallionellaceae* as opposed to a highly significant correlation between relative abundances of *Zetaproteobacteria* and *Gallionellaceae*, providing strong evidence for *Gallionellaceae* being an intrinsic part of the microbial communities in the iron mats.

Numerous hydrothermal systems hosting iron mats containing biogenic stalks have been reported along mid-ocean spreading ridges [54–60]. Nevertheless, extensive studies on community level have been limited [14]. Given the large variability in the overall community structure observed so far in only a few of these mats (this study, [14, 17]), we are most likely only beginning to grasp the full diversity of iron mats in these settings, as well as how they form and what roles they play in elemental cycling.

### Interactions between *Zetaproteobacteria* and *Nitrosopumilus*

In general, little is known about the interactions between iron oxidizers and co-occurring taxa in iron mats, particularly when it comes to *Archaea*. In the iron mats of TWVF, *Zetaproteobacteria* consistently co-occurred with close relatives of ammonium-oxidizing *Thaumarchaeota* within the *Nitrosopumilus* genus (Figs 2 and 4). Genomic analyses have revealed that *Zetaproteobacteria* frequently have genes involved in assimilatory or dissimilatory reduction of nitrate or nitrite, such as nitrate reductase and nitrite reductase [61]. Based on the wide distribution of *nirK* genes, encoding nitrite reductase, in iron mats of the Loihi Seamount, it was recently proposed that the habitat range of *Zetaproteobacteria* includes anoxic habitats containing nitrite [46, 62]. From an analysis of a single iron mat from TWVF, we have already proposed that there might be a link between ammonium-oxidizing *Thaumarchaeota* within the *Nitrosopumilus* genus and iron-oxidizing *Zetaproteobacteria*, whereby nitrite produced by *Nitrosopumilus*, can be used by *Zetaproteobacteria* in anaerobic respiration [17]. In the present study we show that a co-existence between members of *Zetaproteobacteria* and *Nitrosopumilus* is a common feature of iron mats in TWVF, in both the rift valley as well as the rift margin. This opens for the possibility that *Nitrosopumilus* assist in the formation of iron mats and sustain *Zetaproteobacteria* communities in the entire area. Alternatively, members of *Nitrosopumilus* can get entrapped in the iron mats during water circulation. Such entrapment has previously been suggested for explaining the presence of planktonic psychrophiles in a hydrothermal vent ecosystem at Loihi Seamount [63]. In order to understand the relationship between *Nitrosopumilus* and *Zetaproteobacteria* in more detail, it would be interesting to measure *in situ* metabolic rates in addition to doing more comprehensive genomic studies involving the reconstruction of near full-length genomes.

### Shifts in microbial communities and energy densities from the rift margin to the rift valley

*Zetaproteobacteria* seem to depend on Fe(II) as an electron donor [14]. However, although Fe(II) concentrations in a given habitat may be sufficient to support a population of *Zetaproteobacteria*, a number of other factors may control their relative abundance and what organisms they co-occur with. Thermodynamic modelling provides evidence that, on a functional level, shifts in community structures within and between hydrothermal systems can be explained by shifting chemical energy landscapes [1]. With this in mind, it is interesting to note how observed shifts in community composition (Figs 2 and 4, S1 Fig) are co-occurring with shifts in geochemical setting. Specifically, hydrothermal fluids flowing through sediments at the base of the hydrothermal chimneys can be expected to have the same source as the fluids emitted through the chimneys, which have much higher concentrations of H_2_S and methane, than Fe(II) (S1 Table) [1, 64]. At the seafloor the fluids are highly diluted by seawater, and community composition modelling, based on thermodynamic considerations, predict that the communities growing on these fluids will be dominated by sulphide oxidizers and, to a lesser extent, methane oxidizers [1]. This is largely consistent with observed community structures in white mats growing in the vicinity of high-temperature venting chimneys (S1 Fig). Furthermore, analyses of rare-earth elements indicate that the fluid flow in the central rift valley is disconnected from the high-temperature fluids venting through the chimneys, and belong to a separate, more shallow circulation system [17]. As a result, sulphate from seawater is not reduced to sulphide, H_2_S/Fe(II) ratios are low, and energy densities are more dominated by iron oxidation (Fig 6, S1 Table). The composition of rare-earth elements in the diffuse venting fluids of the rift margin indicate that these fluids have the same source as the high-temperature fluids (unpublished data). Unfortunately, we lack data on H_2_S concentrations for these samples. However, the temperature gradients in this region are less steep than at the base of the chimneys (S1 Table), indicating lower fluxes of hydrothermal fluids. Most of the sulphide may therefore have had time to precipitate within the subseafloor as metal sulphides, diminishing the substrate availability for sulphide oxidizers in the iron mats without removing all of the dissolved Fe(II). Yet, energy landscapes seem to be mostly influenced by degradation of organic matter where methanogens consume hydrogen or acetate, presumably produced by fermentative organisms, to produce methane, which in turn is consumed by aerobic methanotrophs (Fig 6). Taken together, microbial mats of the rift valley and the rift margin have largely different community structures (Figs 3 and 4), which seem to reflect that they reside under very different energetic settings. Arguably, obtaining spatially high-resolution data on geochemical settings and distribution of Fe(II) within hydrothermal systems, is key to our understanding of why *Zetaproteobacteria* are distributed the way they are and what taxa and functional groups they co-occur with.

## Conclusion

This study demonstrates that members of *Zetaproteobacteria* are widespread throughout the TWVF, comprising all ZetaOtus considered as cosmopolitan (i.e. ZetaOtus 1,2 and 9). We found no evidence for the presence of abundant Zetaproteobacterial OTUs, specific for TWVF. Relative abundances of *Zetaproteobacteria* and their co-occurring taxa vary considerably between different iron mats. This also seems to include members of iron-oxidizing *Gallionellaceae*. Microbial communities in iron mats from TWVF appear to show a large beta-diversity, which may well be revealed to be even larger if more samples are analysed in the future. Hence, the TWVF seems to be an excellent natural laboratory for investigating the general factors that control the structure and diversity of iron mat microbial communities. Future studies of iron mats from TWVF, involving hydrothermal flow rate measurements, cultivation, shotgun metagenomics studies, and more extensive chemical analyses, seem to be promising approaches in this respect. Nevertheless, building on previous work on linkages between chemical energy and community structure [1], this study provides further evidence that variability in energy density is a key factor shaping microbial communities, including those in iron mats, within and between hydrothermal systems.

## Supporting information

S1 Fig Distribution of inferred functional groups within microbial mats in the TWVF. A white microbial mat, dominated by members of *Sulfurimonas* and growing on the base of a hydrothermal chimney [18], is included in addition to the iron mats analysed in the current study. See also S3 Table for details about assignments of functional groups for dominating OTUs.
(TIF)

S2 Fig Rarefaction curves of iron mat samples and background seawater. Iron mat communities from the rift valley are shown in shades of purple (11ROV6), blue (12ROV9) and green (14ROV13), whereas iron mats from the more active venting sites of the rift margin are shown in shades of red (11ROV3) and orange (12ROV5). The background seawater sample is shown in black.
(TIF)

S3 Fig NMDS plot of the iron mat communities on OTU level. Blue squares indicate samples from the rift valley and red triangles indicate samples from the rift margin. Dots indicate major microbial OTUs and have a radius proportional to overall relative abundance.
(TIF)

S4 Fig Scatterplots showing the relationship between relative abundances of *Gallionellaceae* and *Mariprofundaceae*. (A) Relative abundances of *Gallionellaceae* and total concentration of DNA. (B) A significant positive pearson correlation (R^2^ = 0.7845, p = 0.0008917) was found between relative abundances of *Gallionellaceae* and *Mariprofundaceae*. (B) No correlation was observed between relative abundances of *Gallionellaceae* and DNA concentration (R^2^ = -3.097E-5, p = 0.9429).
(TIF)

S1 Table Overview of the 13 iron mat samples and background seawater and corresponding chemical metadata. T indicates temperatures measured at a depth of approximately 3 cm into the iron mat. Alk = alkalinity, N_reads_ = number of processed high-quality reads, C = Good´s Coverage, S_obs_ = number of different OTUs on 97% identity level, ND = not detected, NA = not analysed.
(DOCX)

S2 Table Processing details of the sequencing data from the 13 iron mat samples and background seawater sample. (DOCX)

S3 Table Closest relatives of the 50 most abundant OTUs (>0.05% total relative abundance) and assignments to metabolic functional groups. (DOCX)
