# Supplementary figures and images for: Microbial analysis of Zetaproteobacteria and co-colonizers of iron mats in the Troll Wall Vent Field, Arctic Mid-Ocean Ridge
Source: PLoS One. 2017 Sep 20;12(9):e0185008. doi: 10.1371/journal.pone.0185008 (PMC5607188; doi:10.1371/journal.pone.0185008)

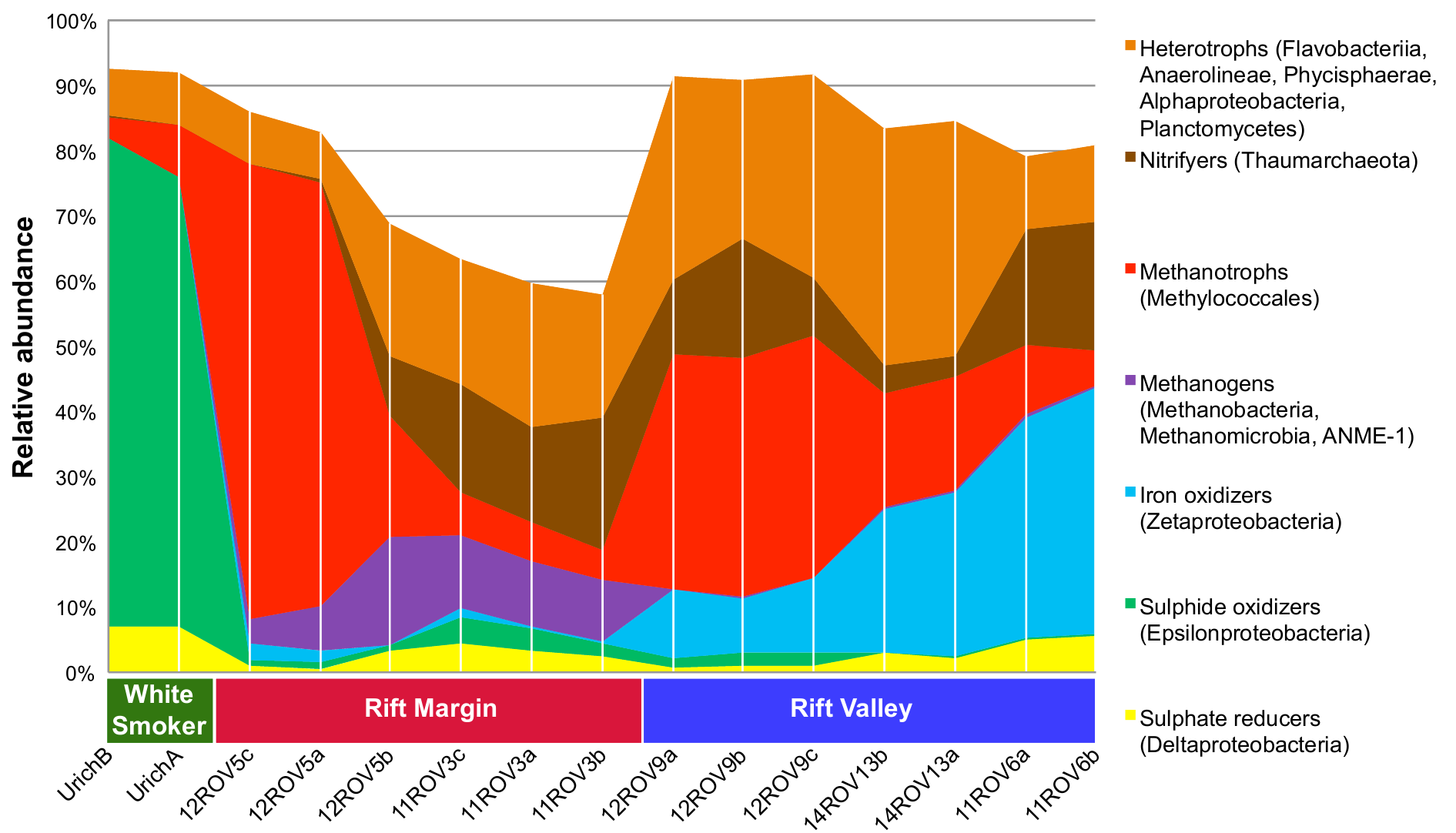

Supplement: S1 Fig — A white microbial mat, dominated by members of Sulfurimonas and growing on the base of a hydrothermal chimney [18], is included in addition to the iron mats analysed in the current study. See also S3 Table for details about assignments of functional groups for dominating OTUs. (TIF) [file pone.0185008.s001.tif]

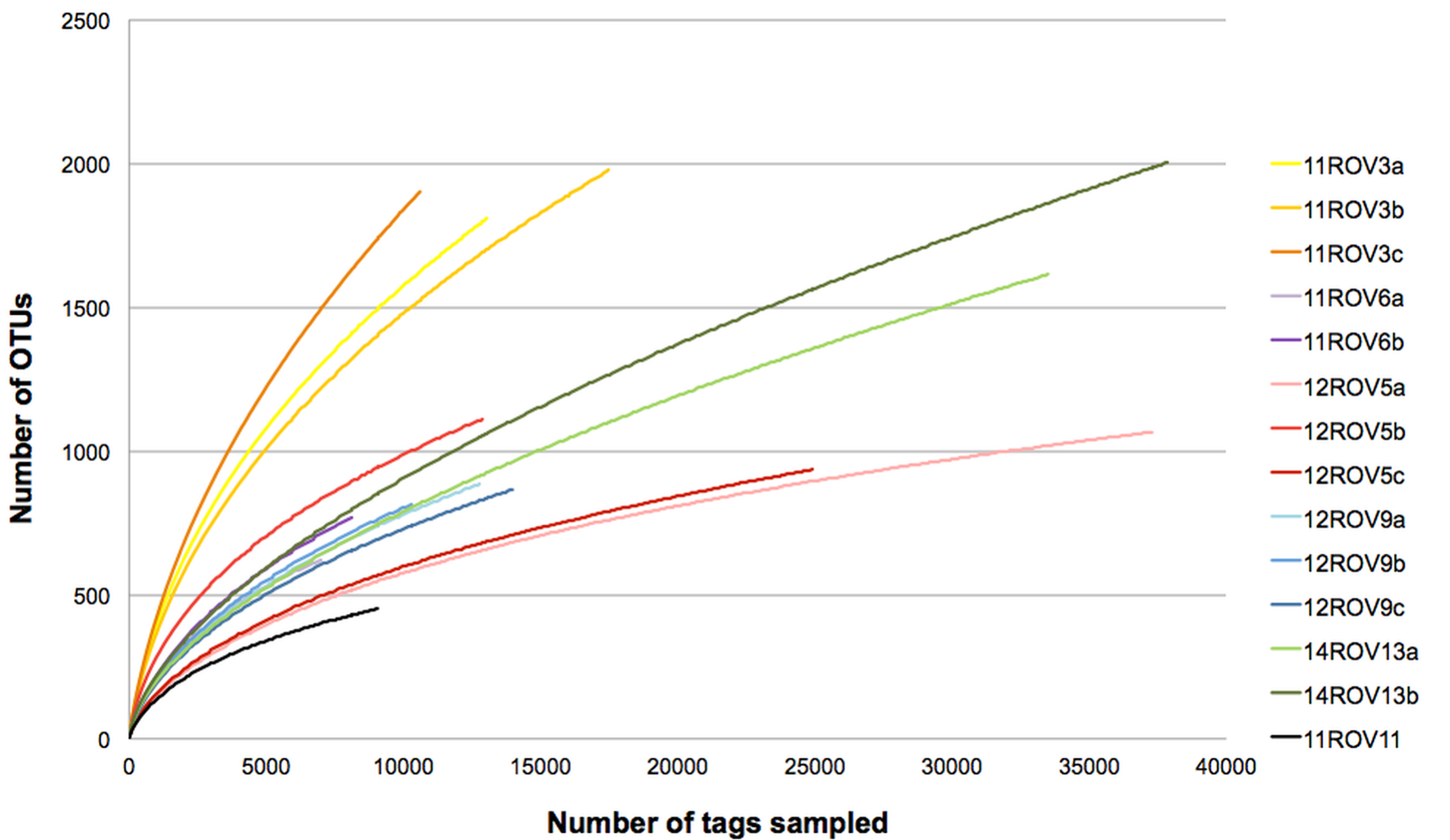

Supplement: S2 Fig — Iron mat communities from the rift valley are shown in shades of purple (11ROV6), blue (12ROV9) and green (14ROV13), whereas iron mats from the more active venting sites of the rift margin are shown in shades of red (11ROV3) and orange (12ROV5). The background seawater sample is shown in black. (TIF) [file pone.0185008.s002.tif]

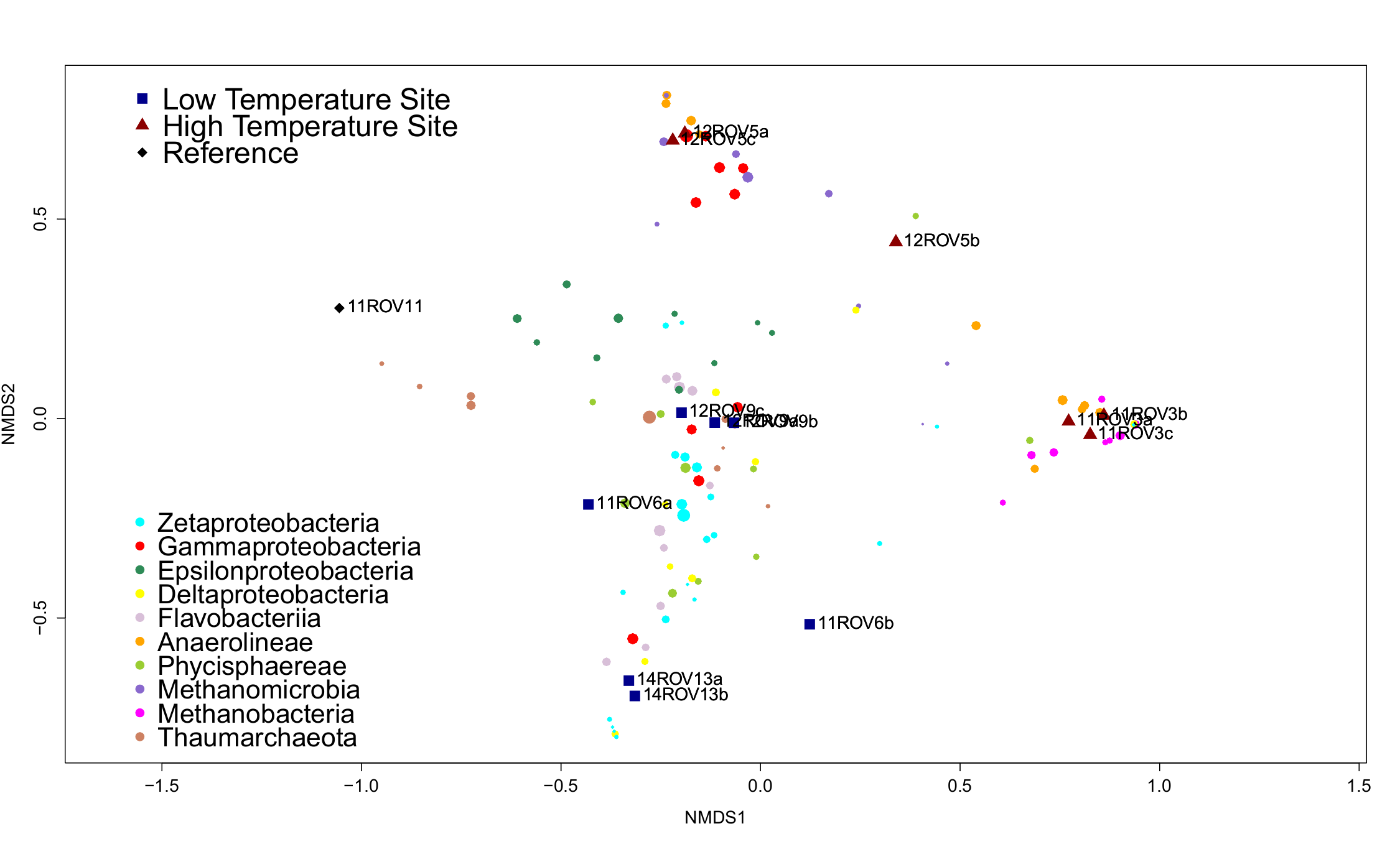

Supplement: S3 Fig — Blue squares indicate samples from the rift valley and red triangles indicate samples from the rift margin. Dots indicate major microbial OTUs and have a radius proportional to overall relative abundance. (TIF) [file pone.0185008.s003.tif]

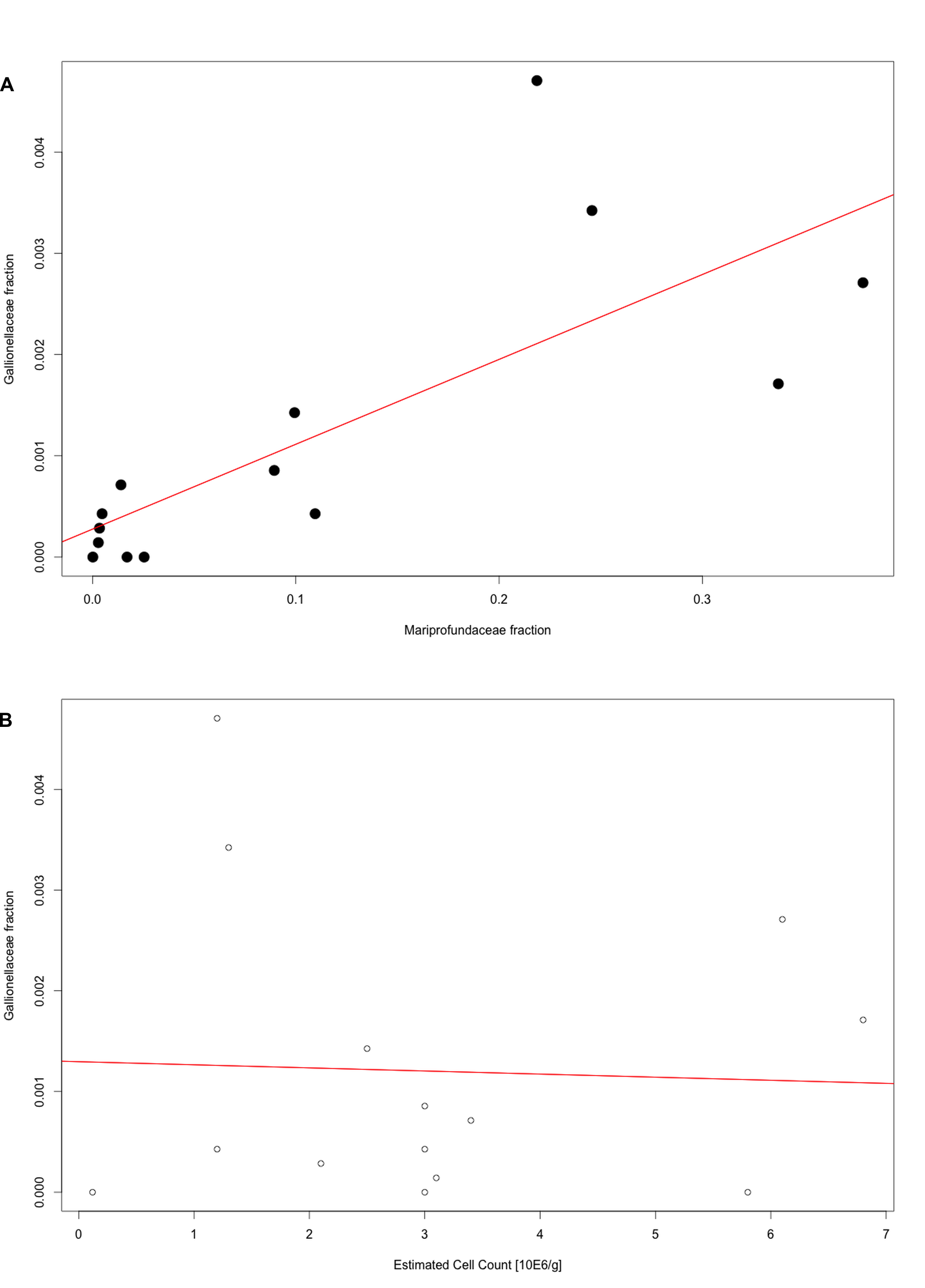

Supplement: S4 Fig — (A) Relative abundances of Gallionellaceae and total concentration of DNA. (B) A significant positive pearson correlation (R2 = 0.7845, p = 0.0008917) was found between relative abundances of Gallionellaceae and Mariprofundaceae. (B) No correlation was observed between relative abundances of Gallionellaceae and DNA concentration (R2 = -3.097E-5, p = 0.9429). (TIF) [file pone.0185008.s004.tif]
