# Supplementary material for: Microbial analysis of Zetaproteobacteria and co-colonizers of iron mats in the Troll Wall Vent Field, Arctic Mid-Ocean Ridge
Source: PLoS One. 2017 Sep 20;12(9):e0185008. doi: 10.1371/journal.pone.0185008 (PMC5607188; doi:10.1371/journal.pone.0185008)
Supplement: S1 Table — T indicates temperatures measured at a depth of approximately 3 cm into the iron mat. Alk = alkalinity, Nreads = number of processed high-quality reads, C = Good´s Coverage, Sobs = number of different OTUs on 97% identity level, ND = not detected, NA = not analysed. (DOCX) [file pone.0185008.s005.docx]

| TWVF  Site | ROV dive | T (°C) | Sampling  year | pH | Alk. | Fe^2+^  [μM] | SO_4_^2-^ [mM] | NH_4_^+^ [μM] | NO_3+2_ [μM] | PO_4_^3-^ [μM] | Sample | Estimated Cell Count* [g^-1^] | N_Reads_ | C | S_obs_ |
| --- | --- | --- | --- | --- | --- | --- | --- | --- | --- | --- | --- | --- | --- | --- | --- |
| Rift Margin | 11ROV3 | 26 | 2011 | 7.21 |  | 7.45 | 28.4 | ND | 6.68 | 0.76 | 11ROV3a | 1.2×10^6^ | 10800 | 0.930 | 1879 |
|  |  |  |  |  |  |  |  |  |  |  | 11ROV3b | 2.1×10^6^ | 13031 | 0.944 | 2052 |
|  |  |  |  |  |  |  |  |  |  |  | 11ROV3c | 3.4×10^6^ | 18060 | 0.903 | 1921 |
|  | 12ROV5 | 85 | 2012 | 7.00 | 2.55 |  |  |  |  | 0.20 | 12ROV5a | 6.8×10^6^ | 50160 | 0.982 | 2085 |
|  |  |  |  |  |  |  |  |  |  |  | 12ROV5b | 6.1×10^6^ | 24962 | 0.981 | 980 |
|  |  |  |  |  |  |  |  |  |  |  | 12ROV5c | 5.8×10^6^ | 18925 | 0.995 | 2548 |
| Rift Valley | 11ROV6 | 5 | 2011 | 7.55 | 2.23 | 144 | 29.4 | 0.44 | ND | 0.12 | 11ROV6a | 3.1×10^6^ | 8334 | 0.950 | 650 |
|  |  |  |  |  |  |  |  |  |  |  | 11ROV6b | 3.0×10^6^ | 6334 | 0.948 | 854 |
|  | 12ROV9 | 2.5 | 2012 | 7.90 | 2.35 |  | 29.7 | 0.80 | 13.15 | 0.10 | 12ROV9a | 2.5×10^6^ | 10514 | 0.952 | 1019 |
|  |  |  |  |  |  |  |  |  |  |  | 12ROV9b | 3.0×10^6^ | 13095 | 0.952 | 947 |
|  |  |  |  |  |  |  |  |  |  |  | 12ROV9c | 3.0×10^6^ | 11731 | 0.962 | 857 |
|  | 14ROV13 |  | 2014 | 7.95 | 2.33 |  |  | ND | 12.12 | 0.82 | 14ROV13a | 1.3×10^6^ | 38955 | 0.967 | 1738 |
|  |  |  |  |  |  |  |  |  |  |  | 14ROV13b | 1.2×10^6^ | 32217 | 0.961 | 2321 |
| Seawater | 11ROV11 |  | 2011 | 7.88 | 2.22 | ND | 29.6 | ND | 5.93 | 0.45 | 11ROV11 | 1.2×10^5^ | 8907 | 0.976 | 450 |

*Estimated as suggested by Button and Robertson, 2001.

Button, D. K. and B. R. Robertson (2001). "Determination of DNA Content of Aquatic Bacteria by Flow Cytometry." *Applied and Environmental Microbiology* **67**(4): 1636-1645.
