# Supplementary material for: Microbial analysis of Zetaproteobacteria and co-colonizers of iron mats in the Troll Wall Vent Field, Arctic Mid-Ocean Ridge
Source: PLoS One. 2017 Sep 20;12(9):e0185008. doi: 10.1371/journal.pone.0185008 (PMC5607188; doi:10.1371/journal.pone.0185008)
Supplement: S2 Table — (DOCX) [file pone.0185008.s006.docx]

**S2 Table: Processing details of the 454 amplicon pool**

| Total number of 16S rRNA sequenced reads | 536.823 |
| --- | --- |
| Processed high-quality reads (after chimera-removal) | 515,796 |
| Overall coverage | 95.86% |
| Average read length | 328 bp |
| Average reads per sample | 33,551 |
