# Supplementary material for: Microbial analysis of Zetaproteobacteria and co-colonizers of iron mats in the Troll Wall Vent Field, Arctic Mid-Ocean Ridge
Source: PLoS One. 2017 Sep 20;12(9):e0185008. doi: 10.1371/journal.pone.0185008 (PMC5607188; doi:10.1371/journal.pone.0185008)
Supplement: S3 Table — (DOCX) [file pone.0185008.s007.docx]

| OTU | % (Relative abundance) | Closest relative (Isolate or strain) | Accession number | Sequence identity | Associated metabolism* |
| --- | --- | --- | --- | --- | --- |
| OTU001 | 9.49 | *Methylococcaceae bacterium* SF-BR | AB453959.1 | 98% | Methanotroph |
| OTU002 | 8.41 | *Nitrosopumilus maritimus* strain NAOA6 | KT380502.1 | 100% | Nitrifyer |
| OTU003 | 7.50 | *Zeta proteobacterium* CSS-1 | KX078246.1 | 99% | FeOB |
| OTU004 | 2.78 | *Pseudofulvibacter sp.* MEBiC08749 | KJ918743.2 | 97% | Heterotroph |
| OTU005 | 2.10 | *Polaribacter sp.* PAMC 27100 | KJ475157.1 | 100% | Heterotroph |
| OTU006 | 2.10 | Endosymbiont of *Alviniconcha sp.* type 1 | AB235238.1 | 92% | Methanotroph? |
| OTU007 | 1.95 | *Marinobacter sp.* 324-2 | HM000083.1 | 97% | ? |
| OTU008 | 1.89 | *Methylococcaceae bacterium* SF-BR | AB453959.1 | 97% | Methanotroph |
| OTU009 | 1.59 | *Methanosaeta pelagica* strain 03d30g | KT189183.1 | 91% | Methanotroph |
| OTU010 | 1.55 | *Methylococcaceae bacterium* SF-BR | AB453959.1 | 98% | Methanotroph |
| OTU011 | 1.55 | *Methylococcaceae bacterium* SF-BR | AB453959.1 | 97% | Methanotroph |
| OTU012 | 1.49 | *Zeta proteobacterium* CSS-1 | KX078246.1 | 98% | FeOB |
| OTU013 | 1.48 | *Methyloprofundus sedimenti* strain WF1 | KF484906.1 | 100% | Methanotroph |
| OTU014 | 1.23 | *Olavius algarvensis* gamma 3 endosymbiont | AJ620496.1 | 100% | SOB |
| OTU015 | 1.01 | *Planctomycete* GMD14H07 | AY162124.1 | 94% | Heterotroph |
| OTU016 | 0.99 | *Methylomonas lenta* strain R-45377 | NR133783.1 | 97% | Methanotroph |
| OTU017 | 0.98 | *Pseudoruegeria sp.* a4 | JX624258.1 | 94% | ? |
| OTU018 | 0.83 | *Zeta proteobacterium* CSS-1 | KX078246.1 | 92% | FeOB |
| OTU019 | 0.81 | Marine gamma proteobacterium HTCC2246 | AY386337.1 | 100% | Heterotroph |
| OTU020 | 0.74 | *Litorilinea aerophila* strain PRI-4131 | NR132330.1 | 88% | Heterotroph |
| OTU021 | 0.71 | Endosymbiont of *Ridgeia piscesae* clone c44 | DQ660822.1 | 98% | SOB |
| OTU022 | 0.62 | Unidentified bacterium clone K2-30-19 | AY344412.1 | 95% | Heterotroph |
| OTU023 | 0.62 | *Levilinea saccharolytica* strain KIBI-1 | NR040972.1 | 89% | Heterotroph |
| OTU024 | 0.58 | *Litoreibacter sp.* KC59 | KX197355.1 | 99% | Heterotroph |
| OTU025 | 0.53 | *Sulfurimonas autotrophica* strain OK5 | AB088432.1 | 97% | SOB |
| OTU026 | 0.48 | Gammaproteobacterium ZOCON-F9 | KJ700455.1 | 99% | Heterotroph? |
| OTU027 | 0.46 | *Dehalococcoides sp.* BHI80-52 | AJ431247.1 | 91% | HOB |
| OTU028 | 0.44 | Crenarchaeote SCGC AAA003-G15 | HQ675748.1 | 100% | Methanogen |
| OTU029 | 0.44 | *Zeta proteobacterium* CSS-1 | KX078246.1 | 97% | FeOB |
| OTU030 | 0.43 | *Bacterium* SH4-10 | JQ269257.1 | 95% | Heterotroph |
| OTU031 | 0.42 | *Ralstonia solanacearum* OE1-1 | CP009764.1 | 100% | Heterotroph |
| OTU032 | 0.41 | *Flavobacterium sp.* CC4V | KM187268.1 | 98% | Heterotroph |
| OTU033 | 0.41 | *Pycnococcus provasolii* strain RCC885 | LN735480.3 | 87% | Heterotroph |
| OTU034 | 0.40 | *Methanothermus sociabilis* strain Kf1-FI | NR104879.1 | 82% | Methanotroph |
| OTU035 | 0.39 | *Bacterium* YC-LK-LKJ27 | KP174640.1 | 87% | ? |
| OTU036 | 0.38 | *Tepidisphaera mucosa* strain 2813 | KM052380.1 | 91% | Heterotroph |
| OTU037 | 0.37 | *Sulfitobacter sp.* NSA 11 | KP204143.1 | 100% | SOB |
| OTU038 | 0.36 | *Antarctobacter sp.* R6a | KT461665.1 | 100% | Heterotroph? |
| OTU039 | 0.35 | *Flavobacteriaceae* bacterium HC2-3 | JF312946.1 | 99% | Heterotroph |
| OTU040 | 0.35 | *Acidimicrobidae* bacterium YM18-244 | AB360344.1 | 97% | Heterotroph, FeoB? |
| OTU041 | 0.34 | *Chloroflexi* bacterium JGI 0000059-C09 | KJ535426.1 | 90% | Heterotroph |
| OTU042 | 0.34 | *Sagittula marina* strain F028-2 | NR109096.1 | 99% | Heterotroph |
| OTU043 | 0.33 | Bacterium episymbiont of *Kiwa sp.* clone ARB D3684AA7 | KF439043.1 | 97% | SOB? |
| OTU044 | 0.33 | *Alpha proteobacterium* PR-2 | KJ442652.1 | 95% | Heterotroph |
| OTU045 | 0.33 | *Thermanaerothrix daxensis* strain GNS-1 | NR117865.1 | 91% | Heterotroph |
| OTU046 | 0.32 | Endosymbiont of *Ridgeia piscesae* clone HF8M1b2 | JX570607.1 | 100% | SOB |
| OTU047 | 0.32 | *Methanosaeta sp.* Mx | KP205578.1 | 93% | Methanotroph |
| OTU048 | 0.31 | *Planctomycete* GMD14H07 | AY162124.1 | 95% | Heterotroph |
| OTU049 | 0.31 | *Alpha proteobacterium* dMB-MAT36 | AB458529.1 | 99% | Heterotroph |
| OTU050 | 0.30 | *Methanobacterium bryantii* culture DSM:862 | JQ346748.1 | 84% | Methanotroph |

**S3 Table: Closest relative and associated metabolism of the 50 most common OTUs at TWVF**

* Metabolism of closest relative (isolate/strain) as reported. (FeOB = (Neutrophillic) iron oxidizer, SOB = Sulfide oxidizer, SRB = sulphate reducer, HOB = hydrogen oxidizer.)
